# Differences in change of post‐operative antioxidant levels between laser‐assisted lenticule extraction and femtosecond laser in situ keratomileusis

**DOI:** 10.1111/jcmm.18069

**Published:** 2023-12-05

**Authors:** Hung‐Chi Chen, Shun‐Fa Yang, Chia‐Yi Lee, Yi‐Jen Hsueh, Jing‐Yang Huang, Chao‐Kai Chang

**Affiliations:** ^1^ Department of Ophthalmology Chang Gung Memorial Hospital Linkou Taiwan; ^2^ Department of Medicine Chang Gung University College of Medicine Taoyuan Taiwan; ^3^ Center for Tissue Engineering Chang Gung Memorial Hospital Linkou Taiwan; ^4^ Institute of Medicine, Chung Shan Medical University Taichung Taiwan; ^5^ Department of Medical Research Chung Shan Medical University Hospital Taichung Taiwan; ^6^ Nobel Eye Institute Taipei Taiwan; ^7^ Department of Ophthalmology, Jen‐Ai Hospital Dali Branch Taichung Taiwan; ^8^ Department of Optometry Da‐Yeh University Chunghua Taiwan

**Keywords:** ascorbic acid, dry eye disease, femtosecond laser in situ keratomileusis, laser‐assisted lenticule extraction, total antioxidant capacity

## Abstract

To evaluate the change of total antioxidant capacity (TAC) and ascorbic acid (AA) between femtosecond laser in situ keratomileusis (FS‐LASIK) and laser‐assisted lenticule extraction (LALEX). A prospective non‐randomized study was conducted, and 33 and 75 eyes that had undergone FS‐LASIK or LALEX surgeries were enrolled, respectively. The tear films near corneal incisions were collected, and the concentrations of TAC and AA were determined. The generalized linear mixed model was adopted to calculate the adjusted odds ratio (aOR) with 95% confidence interval (CI) of TAC and AA between the two groups. The AA reduction was significant 1 month after the LALEX and FS‐LASIK procedures (both *p* < 0.05), and the decrement in AA level was significantly larger in the FS‐LASIK group compared to the LALEX group (*p* = 0.0002). In the subgroup analysis, the LALEX group demonstrated a lower decrement in TAC level in the individuals with dry eye disease (DED) than the FS‐LASIK group (*p* = 0.0424), and the LALEX group demonstrated a significantly lower AA decrement in the participants with high myopia (*p* = 0.0165) and DED (*p* = 0.0043). The LALEX surgery causes lesser AA decrement compared to FS‐LASIK surgery especially for the patients with DED.

## 1 INTRODUCTION

The keratorefractive surgeries for myopia, hyperopia and astigmatism corrections have gained popularity in the past decades, and multiple types of surgery are available now.^1^ The main types of corneal refractive surgery include photorefractive keratectomy, microkeratome laser in situ keratomileusis, femtosecond laser in situ keratomileusis (FS‐LASIK) and the laser‐assisted lenticule extraction (LALEX),^2^ and the numbers of patients receiving FS‐LASIK and LALEX have gradually increased.^3^ The visual and refractive outcomes of FS‐LASIK and LALEX were similar, while the post‐operative corneal sensitivity was greater in the LALEX procedure according to a previous publication.^4^ On the other hand, the optical density, which may cause foggy vision, was higher in the individuals who received LALEX surgery during the early post‐operative periods.^5^

Although the safety of FS‐LASIK and LALEX is guaranteed, there are still some post‐operative complications that can disturb visual acuity and quality.^6^ Dry eye disease (DED), which can influence the visual acuity, is the most common post‐operative complications of refractive surgeries including FS‐LASIK and LALEX.^2^, ^7^, ^8^ FS‐LASIK shows a higher rate of severe DED compared to LALEX based on the subjective symptoms and the tear break‐up time, while severe DED can occur in individuals who have received LALEX surgery.^9^ In addition, the post‐operative corneal wound healing process is a major issue in the FS‐LASIK and LALEX, which delays corneal healing and causes irritation and decreased vision.^10^ Regarding the severe complications, the diffuse lamellar keratitis and infectious keratitis can lead to prominent visual impairment in individuals received refractive surgeries.^6^ Besides, the corneal endothelial damage after refractive surgery was reported, although the incidence was low.^11^

The expression of oxidative stress is crucial in several systemic and ocular diseases.^12^, ^13^ Patients with DED demonstrated a higher oxidative stress and inflammatory response in a previous study.^14^ Besides, the alkali burn of cornea can be alleviated by the application of N‐acetyl‐L‐cysteine and ascorbic acid (AA) in experimental and clinical research, respectively.^15^, ^16^ However, few studies have discussed the change of antioxidants, including the total antioxidant capacity (TAC) and AA, after the refractive surgeries. Because the laser application during the refractive surgeries can elevate the inflammation, which can be related to oxidative stress,^17^, ^18^ an alteration of post‐operative TAC and AA may be possible. Moreover, the change of antioxidants could be different between FS‐LASIK and LALEX since different lasers are applied in the two surgeries, which requires further investigation.

Accordingly, the purpose of the present study is to evaluate the concentrations of TAC and AA before and after refractive including the FS‐LASIK and LALEX procedures. Besides, whether the differences of TAC and AA alterations between the two refractive surgeries were also analysed in the multivariable model.

## 2 MATERIALS AND METHODS

### 2.1 Participant selection

A prospective non‐randomized study was executed in the Nobel Eye Clinic, Taipei branch, which is an institution specialized in cataract and refractive surgeries. The participants enrolled in the present study met the following criteria: (1) age between 20 and 50 years, (2) the existence of myopia for at least −1.00 diopter (D), (3) participant can understand all details of the present study. Besides, the following exclusion criteria were applied to maintain the homogeneity of the study population: (1) un‐corrected visual acuity (UCVA) lower than counting finger level, (2) the existence of any types of cataract, (3) the existence of clinical significant or subclinical keratoconus and other corneal ectasic disorders, (4) the existence of severe retinal disease like proliferative diabetic retinopathy retinal detachment, vitreous haemorrhage and macular pucker, (5) the existence of any types of end‐stage glaucoma, (6) the existence of severe DED, central corneal scars, active corneal erosions and corneal neovascularization, (7) the existence of previous eyeball rupture and ptosis that cover the pupil, (8) the existence of optic nerve atrophy or ischemic optic neuropathy, (9) unstable refractive status with the progression of any refractive error of more than 0.5 D in the past 2 years, (10) pregnant status and (11) active inflammatory diseases, including the systemic lupus erythematous, diabetes mellitus, thyroid disease, Sjogren syndrome, rheumatic arthritis, ankylosing spondylitis and systemic sclerosis. After the whole selection process, 75 eyes from 38 participants and 33 eyes from 17 participants were categorized into the LALEX group and FS‐LASIK group, respectively. These participants were analysed in the multivariable model with adjustments of several covariates including age and sex.

### 2.2 Surgical technique

The LALEX and FS‐LASIK procedures in the present study were performed by one experienced refractive specialist (C.‐K.C.). The LALEX procedure was done using one femtosecond laser device (Visuamax 500, Carl Zeiss, Göschwitzer Str.). The optic zone was set as 5.5–6.9 mm based on the pupil size and ablation depth, and the incision was 3.0 mm and created at 105 degrees. After the corneal apex was confirmed manually by microscope with the assistance of topography and corneal reflex, the cornea was fixed by the suction device, and the corneal apex was put in the centre of suction ring. After the strike of the femtosecond laser, a spatula was used to dissect the lenticule, which was then extracted by a forceps. The FS‐LASIK was performed by one femtosecond laser device (Visuamax 500, Carl Zeiss, Göschwitzer Str.) and an excimer laser device (EX500, Alcon Laboratories). After fixing the cornea with the suction device, the femtosecond laser device created a corneal flap with diameters from 7.5 to 8.5. Then, the corneal flap was lifted by a spatula, and the excimer laser was emitted after the registration of iris centre by the excimer laser device. Upon the completion of excimer laser emission, the stromal bed was irrigated by normal saline, and the corneal flap was placed at the original site. A soft contact lens was applied to cover the corneal surface then removed 1 h after the FS‐LASIK procedure.

### 2.3 Ophthalmic examination

All the participants received identical examinations in our clinic. The pre‐operative examinations included UCVA, best‐corrected visual acuity (BCVA), intraocular pressure (IOP) with central corneal thickness (CCT) measurement via pneumatic tonometry (NT‐530, NIDEK), cyclopegic refraction of sphere as well as cylinder power via autorefractor (KR‐8900, Topcon), corneal curvature and apex by topographic device (Oculus Pentacam, OCULUS Optikgeräte GmbH) and Schirmer *I* test (the Schirmer strip was applied after topical anaesthesia, and the participants closed their eyes for 5 min; the length of the wet portion was documented after the removal of the Schirmer strip). The post‐operative examinations included UCVA, IOP, sphere power and cylinder power which were accessed via the identical devices. The data before the surgery, 1 week after the surgery and 1 month after the surgery were obtained and analysed.

### 2.4 Antioxidant agent determination

The collection and analysis of TAC and AA data were executed with the similar procedures described in our previous publications.^19^, ^20^ The tear film was collected pre‐operatively, 1 week post‐operatively and 1 month post‐operatively using the Schirmer strip. At the above time points, the corneal epithelial surface was intact and thus avoid the influence of antioxidant that released from the incision during the early post‐operative period of refractive surgery. We collected the tear sample at the superior‐temporal site (between 10 and 11 clock hours) of right eye and superior‐nasal site (around 1 clock hour) of the left eye, which are sites near the incision of both FS‐LASIK and LALEX. The Schirmer strip was placed at the conjunctiva near the limbal region of corneal incision in FS‐LASIK or LALEX for 5 min, then the tear film sample in the Schirmer strip was transported in liquid nitrogen (−196°C) and stored at −80°C. The determination of the tear film's TAC and AA concentrations was executed using the colorimetric OxiselectTM Ascorbic Acid Assay Kit (FRASC, Cell Biolabs, Inc.), which is in accordance with the reduction in ferric ions through the applications of ascorbic oxidase. Before the analysis, tear film samples were thawed to ~4°C. The weight of each Schirmer strip containing tear film was measured to obtain the weight of tear film, and the Schirmer strip was immersed into the biomasher and diluted by the colorimetric assay buffer to a one‐tenth concentration for 5 min. After that, the solution was centrifuged with 12,800 relative centrifugal force (RCF) for 5 min under 4°C, and the centrate was collected and centrifuged with 12,800 RCF for another 5 min under 4°C. Then the colorimetric measurements were done within a 540–600 nm wavelength via an absorbance microplate reader (SunriseTM Tecan), and the levels of TAC and AA in the centrifuged sample were defined. Importantly, the quantification of TAC and AA levels was demonstrated in units of millimoles per litre (mmol/L or mM). To ensure the precision of each tear film sample, all the tear film samples were measured three times, and the average amount of TAC and AA concentrations was collected and put into following analysis.

### 2.5 Statistical analysis

The SPSS version 20.0 (SPSS Inc.) was utilized for all the statistical analyses in the present study. The Shapiro–Wilk test was applied to examine the normal distribution of LALEX and FS‐LASIK groups, which revealed normal distribution. Descriptive analysis was applied to illustrate the age, sex, systemic disorders and the ocular diseases, then an independent *T* test was adopted to compare these indexes between groups. An independent *T* test was also conducted to analyse the results of pre‐operative and post‐operative ophthalmic examinations between the two groups. For the change of antioxidant agents, a pair‐*T* test was used to compare the TAC and AA amounts before LALEX and FS‐LASIK surgeries and 1 month after refractive surgeries, and an independent *T* test was applied to demonstrate the difference of pre‐operative and post‐operative TAC and AA between the whole LALEX and FS‐LASIK groups, the sex‐wise subgroups and age distribution‐wise subgroups (divided by 35 years old). After the above analyses, the generalized linear mixed model was utilized to investigate the trend of TAC and AA alteration between the two groups with adjustments for age, sex, sphere power, cylinder power, IOP, results of Schirmer test, CCT, optic zone and the baseline TAC or AA levels, and the adjusted odds ratio (aOR) with 95% confidence interval (CI) of TAC and AA of LALEX group compared to FS‐LASIK group was produced. In the subgroup analyses, specific populations in the LALEX and FS‐LASIK groups were defined according to subsequent conditions: high sphere/myopia (higher than −5.00 D sphere power), high astigmatism (higher than −2.00 D cylinder power), the presence of pre‐operative DED (defined by C.‐K.C. according to the result of Schirmer *I* test, tear break‐up time and ocular surface condition) and large optic zone (>7.0 mm). The generalized linear mixed model was employed again for our subgroup analysis to calculate the difference of TAC and AA changes of LALEX group compared to the FS‐LASIK group with the adjustment of age, sex and the baseline TAC or AA levels. A *p* value <0.05 was defined as statistical significant in the present study.

## 3 RESULTS

### 3.1 Demographic data and ophthalmic examinations

The demographic characters of the LALEX and FS‐LASIK groups are demonstrated in Table 1. The mean ages in the LALEX and FS‐LASIK groups were 32.69 ± 6.81 and 35.17 ± 6.76, respectively, showing no significant difference (*p* = 0.1345). The FS‐LASIK group showed a numerically higher male ratio compared to the LALEX group but did not reach statistical significance (12:11 vs 16:32, *p* = 0.0537). Also, there was no significant difference in distribution of systemic diseases and ocular diseases (both groups *p* > 0.05) (Table 1). Before the performance of refractive surgery, the FS‐LASIK group showed a higher sphere power than the LALEX group (−7.56 ± 2.79 vs −5.79 ± 2.02, *p* = 0.0001), while the optic zone in the LALEX group was significantly larger than in the FS‐LASIK group (6.37 ± 0.28 vs 6.54 ± 0.23, *p* = 0.0001). One week post‐operatively, the IOP was significantly higher in the LALEX group than in the FS‐LASIK (15.53 ± 1.90 vs 14.15 ± 1.48, *p* = 0.0001). Besides, the cylinder power was lower in the FS‐LASIK group compared to LALEX group throughout the post‐operative period (both *p* < 0.05) (Table 2).

**TABLE 1:** The baseline characters of the study population.

Character	LALEX group (*N* = 75)	FS‐LASIK group (*N* = 33)	*p* Value
Age (mean ± SD)	32.69 ± 6.81	35.17 ± 6.76	0.1345
Sex (male:female)	16:32	12:11	0.0537
Systemic disease			
Hypertension	0	0	1.0000
Diabetes mellitus	1	1	1.0000
Autoimmune disease	1	0	0.9255
Thyroid disease	2	0	0.9123
Ocular disease	27	12	0.7454

Abbreviations: FS‐LASIK, femtosecond laser‐assisted‐laser in situ keratomileusis; LALEX, laser‐assisted lenticule extraction; *N*, number; SD, standard deviation.

**TABLE 2:** The post‐operative outcome after the refractive surgery.

Parameter	LALEX group (*N* = 75)	FS‐LASIK group (*N* = 33)	*p* Value
Pre‐OP			
BCVA	0.99 ± 0.02	0.98 ± 0.07	0.1666
Sphere	−5.79 ± 2.02	−7.56 ± 2.79	0.0001^a^
Cylinder	−1.06 ± 0.73	−1.43 ± 1.20	0.0590
IOP	16.31 ± 2.36	16.24 ± 2.09	0.8541
Schirmer test	12.64 ± 6.71	14.65 ± 9.25	0.1915
CCT	546.81 ± 28.87	538.57 ± 27.91	0.1107
Optic zone	6.54 ± 0.23	6.37 ± 0.28	0.0001^a^
Post‐OP 1 week			
UCVA	0.94 ± 0.14	0.93 ± 0.12	0.7073
Sphere	−0.06 ± 0.49	0.10 ± 0.90	0.2386
Cylinder	−0.61 ± 0.31	−0.45 ± 0.31	0.0065^a^
IOP	15.53 ± 1.90	14.15 ± 1.48	0.0001^a^
Post‐OP 1 month			
UCVA	0.97 ± 0.12	0.94 ± 0.15	0.2958
Sphere	−0.19 ± 0.50	−0.07 ± 0.85	0.3812
Cylinder	−0.60 ± 0.31	−0.45 ± 0.34	0.0082^a^
IOP	15.59 ± 2.34	14.93 ± 1.73	0.0633

Abbreviations: BCVA, best‐corrected visual acuity; CCT, central corneal thickness; FS‐LASIK, femtosecond laser‐assisted‐laser in situ keratomileusis; IOP, intraocular pressure; LALEX, laser‐assisted lenticule extraction; *N*, number; OP, operation; UCVA, un‐corrected visual acuity.

^a^ Denotes significant difference between the two groups.

### 3.2 Concentration of antioxidants between the LALEX and FS‐LASIK groups

**FIGURE 1:**
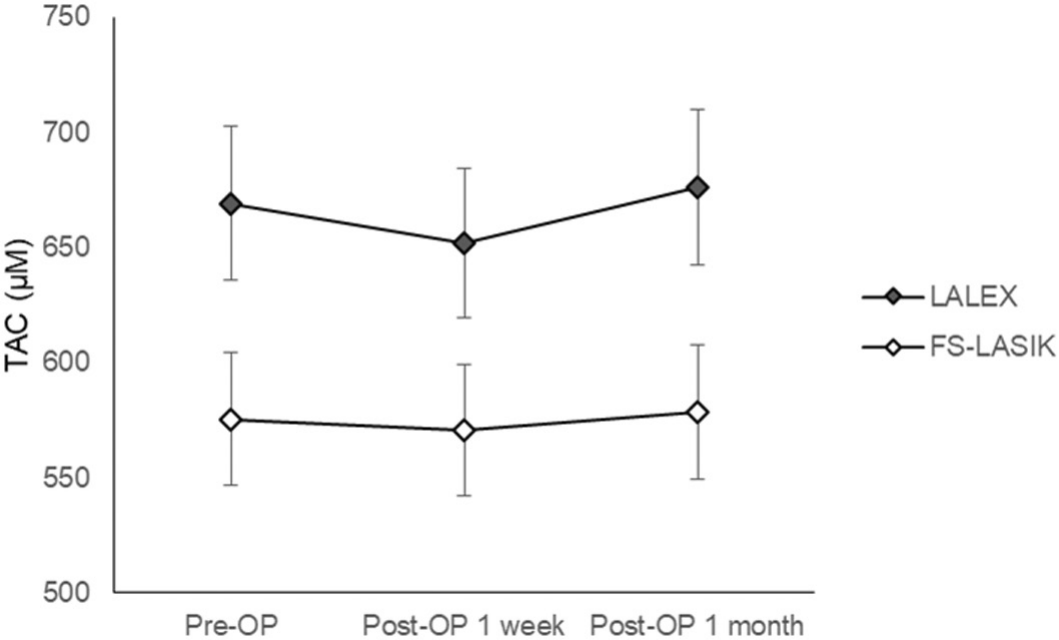
The change of total antioxidant capacity during the follow‐up period between the two groups. FS‐LASIK, femtosecond laser‐assisted‐laser in situ keratomileusis; LALEX, laser‐assisted lenticule extraction; post‐OP, post‐operative; pre‐OP, pre‐operative; TAC, total antioxidant capacity.

**FIGURE 2:**
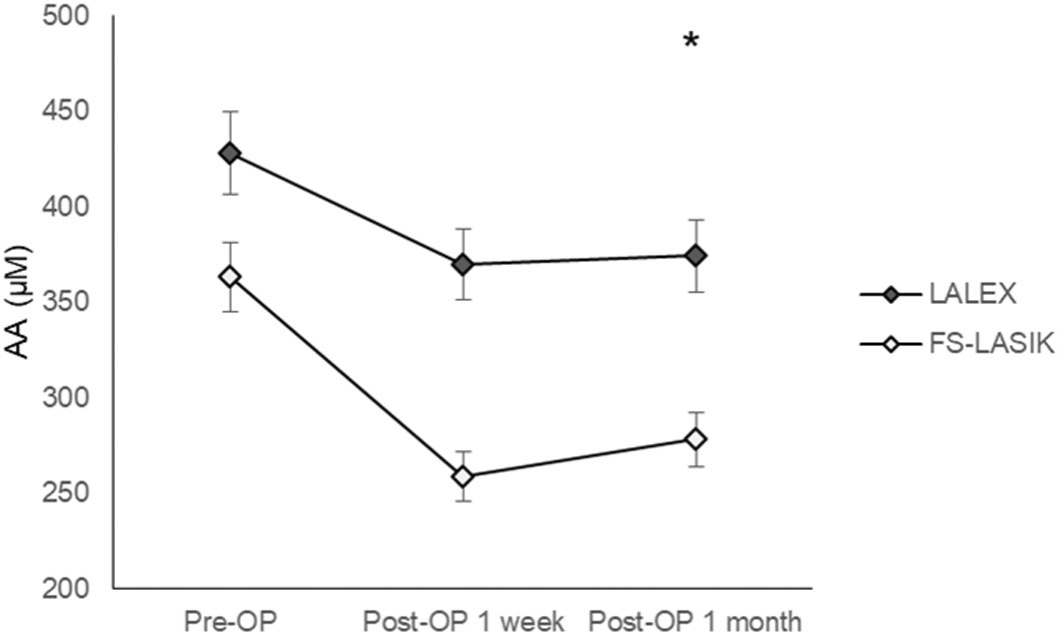
The change of ascorbic acid during the follow‐up period between the two groups. AA, ascorbic acid; FS‐LASIK, femtosecond laser‐assisted‐laser in situ keratomileusis; LALEX, laser‐assisted lenticule extraction; post‐OP, post‐operative; pre‐OP, pre‐operative. *Denotes significant difference between the two groups.

The TAC level 1 month post‐operatively was similar to the pre‐operative value in both the LALEX (676.12 ± 146.13 vs 669.11 ± 261.74, *p* = 0.8813) and FS‐LASIK (578.58 ± 147.44 vs 575.41 ± 387.70, *p* = 0.9017) groups, while the AA reduction was significant 1 month after both LALEX (374.36 ± 117.40 vs 428.16 ± 199.04, *p* = 0.0028) and FS‐LASIK (278.25 ± 178.96 vs 363.29 ± 113.99, *p* = 0.0001) procedures. However, the pre‐operative TAC concentration was significantly higher in the LALEX group compared to the FS‐LASIK group (669.11 ± 261.74 vs 575.41 ± 387.70, *p* = 0.0124) and remained so throughout the study period (both *p* < 0.05) (Table 2). The pre‐operative AA concentration was similar between the LALEX and FS‐LASIK groups (428.16 ± 199.04 vs 363.29 ± 113.99, *p* = 0.5245). However, the AA concentration was significantly higher in the LALEX group than the FS‐LASIK group 1 day post‐operatively (369.54 ± 118.10 vs 258.69 ± 158.67, *p* = 0.0005) and 1 month post‐operatively (374.36 ± 117.40 vs 278.25 ± 178.96, *p* = 0.0019) (Table 3). The sex‐wise and age distribution‐wise subgroup analyses revealed similar results as the whole‐group analysis except the pre‐operative and one‐week post‐operative TAC levels between LALEX and FS‐LASIK surgeries were similar in male population (both *p* > 0.05) (Tables S1 and S2). Regarding the trend of antioxidant changes between the LALEX and FS‐LASIK groups, the change of TAC concentration was similar between the LALEX and FS‐LASIK groups (aOR: 0.996, 95% CI: 0.201–1.857, *p* = 0.6012) (Figure 1). On the other hand, the decrement in AA level during the study interval was significantly lower in the LALEX group compared to the FS‐LASIK group after adjusting for several potential confounders (aOR: 0.799, 95% CI: 0.665–0.854, *p* = 0.0056) (Figure 2).

**TABLE 3:** The level of antioxidant before and after surgery between groups.

Antioxidant	LALEX group (*N* = 75)	FS‐LASIK group (*N* = 33)	*p* Value
TAC			
Pre‐OP	669.11 ± 261.74	575.41 ± 387.70	0.0124^a^
Post‐OP 1 week	652.11 ± 224.16	570.63 ± 225.05	0.0431^a^
Post‐OP 1 month	676.12 ± 146.13	578.58 ± 147.44	0.0254^a^
AA			
Pre‐OP	428.16 ± 199.04	363.29 ± 113.99	0.5245
Post‐OP 1 week	369.54 ± 118.10	258.69 ± 158.67	0.0005^a^
Post‐OP 1 month	374.36 ± 117.40	278.25 ± 178.96	0.0019^a^

Abbreviations: AA, ascorbic acid; FS‐LASIK, femtosecond laser‐assisted‐laser in situ keratomileusis; LALEX, laser‐assisted lenticule extraction; *N*, number; OP, operation; TAC, total antioxidant capacity.

^a^ Denotes significant difference between the two groups.

### 3.3 Subgroup analysis stratified by ophthalmic covariates

In the subgroup analysis, the LALEX group demonstrated a lower decrement in TAC level in the individuals with DED than the FS‐LASIK group (aOR: 0.889, 95% CI: 0.707–0.961, *p* = 0.0449). The LALEX group demonstrated a significantly lower AA decrement in the participants with high sphere/myopia (aOR: 0.840, 95% CI: 0.623–0.962, *p* = 0.0377) and DED (aOR: 0.763, 95% CI: 0.694–0.817, *p* = 0.0035). The other subgroup analyses did not show significant between‐group differences (all *p* > 0.05) (Table 4).

**TABLE 4:** The subgroup analyses stratified by different parameters.

Subgroup	aOR^a^	95% CI	*p* Value
TAC			
High sphere	0.988	0.897–1.141	0.2243
High cylinder	1.228	0.902–1.539	0.3742
DED	0.889	0.707–0.961	0.0449^b^
Large zone	1.006	0.928–1.193	0.7165
AA			
High sphere	0.840	0.623–0.962	0.0377^b^
High cylinder	0.925	0.891–1.037	0.2296
DED	0.763	0.694–0.817	0.0035^b^
Large zone	0.984	0.870–1.206	0.6721

Abbreviations: AA, ascorbic acid; aOR, adjusted odds ratio; CI, confidence interval; DED, dry eye disease; *N*, number; OP, operation; TAC, total antioxidant capacity.

^a^ The risk of LALEX procedure compared to FS‐LASIK procedure.

^b^ Denotes significant difference between the two groups.

## 4 DISCUSSION

The oxidative stress and related antioxidant changes can cause the development of several ocular surface and anterior segment diseases.^14^, ^21^, ^22^, ^23^, ^24^, ^25^ The formation of DED is associated with the oxidative stress on the ocular surface in both Sjogren and non‐Sjogren syndrome‐related DED.^24^ The biomarker of oxidative stress including the 4‐hydroxynonenal and malondialdehyde was observed to increase in the ocular surface and tear film in patients with DED compared to the general population.^14^ Besides, the application of melatonin can ameliorate the oxidative stress‐associated damage in the DED model.^26^ The corneal diseases, including the keratoconus, corneal fibrosis and Fuchs endothelial corneal dystrophy, can also be influenced by the existence of oxidative stress.^27^, ^28^, ^29^, ^30^ The corneal endothelium of rabbit eye that underwent benzalkonium chloride insult recovered faster with clear corneal appearance under the application of AA compared to a control group.^19^ Moreover, the utilization of AA can lead to the proliferation of human corneal endothelial cells in vitro,^31^ and the instillation of AA perioperatively could reduce the damage to the endothelium results from cataract surgery.^32^ Besides, the proliferative ability of human corneal endothelial cell was demonstrated after the instillation of AA in a case report.^33^ Regarding other ocular diseases that correlate to the elevation of oxidative stress, the TAC in the vitreous cavity and pigment epithelium‐derived factor could serve as protective factors for proliferative diabetic retinopathy.^34^ Furthermore, the formation of cataract resulting from the high oxidative stress after the trans pars plana vitrectomy might be delayed by the application of AA.^35^ The laser refractive surgeries, including the LALEX and FS‐LASIK, remove additional corneal tissue, which can cause the post‐operative inflammation of the residual corneal tissue.^6^ The inflammation and related post‐operative complications, including stromal inflammation and wound healing, are common in the LALEX and FS‐LASIK procedures.^6^ The inflammatory response in cornea could be associated with the increment in oxidative stress and decrement in antioxidant agents.^18^ Because the procedures and laser types of LALEX and FS‐LASIK are different,^17^ we speculate that the change of antioxidant agents may also be different. This speculation was supported by the results of the present study to some extent.

The concentration of AA reduced significantly after either the LALEX or FS‐LASIK surgeries, and the decrement was greater in the latter than the former. On the other hand, the change of TAC after the two refractive surgeries was not significant, and the LALEX and FS‐LASIK surgeries demonstrated similar fluctuations of TAC. In a previous study, the post‐operative inflammation was higher in individuals who underwent FS‐LASIK procedure than the LALEX procedure, while no evaluation of antioxidant change was reported.^17^ To our knowledge, the present study is the first to depict the different antioxidant reductions between the LALEX and FS‐LASIK surgeries. Moreover, we adjusted several demographic data and ophthalmic indexes in the generalized linear mixed model to reduce the influence of other factors on antioxidant alterations. Consequently, the LALEX procedure may indeed achieve better antioxidant preservation than the FS‐LASIK procedure. Regarding the change of AA in other ophthalmic disorders and managements, the concentration of AA is associated with the health status of human corneal endothelial cells.^36^ Moreover, reduction in AA can be observed in patients who received cataract surgery, and the degree of AA decrement correlates to the severity of the cataract.^37^ Thus, the AA may play a crucial role in the modification of ocular oxidative stress. The laser emitted during the LALEX procedure is less than that in the FS‐LASIK procedure because the additional corneal tissue was taken out in LALEX (rather than evaporated by laser in FS‐LASIK).^17^ This may be the reason for our findings, since the thermal effect of laser could lead to the elevation of oxidative stress. In addition, the inflammatory response was significantly higher in the FS‐LASIK than the LALEX which may cause the elevation of oxidative stress^18^, ^38^; thus the inflammatory‐associated oxidative stress could be higher in the FS‐LASIK group. About the similar TAC changes between groups, we hypothesize that the TAC consisted of several antioxidants, including superoxide dismutase and catalase on the corneal surface,^12^, ^39^ and that some antioxidants may not be altered after the LALEX and FS‐LASIK surgeries. Nevertheless, further research is need to clarify this hypothesis.

In the subgroup analyses, the DED individuals received LALEX surgery demonstrated a significantly lower amount of both TAC and AA decrements compared to the DED patients received FS‐LASIK surgery. Furthermore, individuals with high myopia illustrate a significantly higher AA reduction after FS‐LASIK surgery compared to those who received LALEX surgery. There was rare study to discuss the change of antioxidant between LALEX and FS‐LASIK surgeries in specific populations. DED, as we mentioned in previous paragraphs, is a disease with elevated inflammatory response and oxidative stress.^24^ Several medications for DED, including corticosteroid and cyclosporine, target and reduce the inflammatory response in DED and improve symptoms and signs.^40^ On the other hand, the post‐operative DED occurred more commonly in the individuals received FS‐LASIK surgery than the individuals received LALEX surgery due to the degree of corneal denervation.^41^ The subjective symptoms and tear film stability in post‐operative DED require about 6–12 months to recover after the arrangement of FS‐LASIK surgery, but only 3–6 months with LALEX surgery.^9^ Accordingly, the FS‐LASIK procedure may trigger more reduction in antioxidant including the TAC and AA, since the higher laser energy in FS‐LASIK could elevate the inflammation and oxidative stress easily in DED patients, who are vulnerable to inflammation and oxidative stress. Regarding the high myopia group, the prominent decrement in AA in the FS‐LASIK group may result from the higher laser energy applied in the high myopia patients (i.e. to evaporate thicker corneal tissue). Although the total laser energy of LALEX surgery may also be higher in the patients with high myopia, the difference is not as huge as FS‐LASIK surgery, since only few planes are cut during LALEX surgery.^42^ Besides, high myopia patients have a higher baseline oxidative stress and inflammation compared to those with low myopia,^43^, ^44^, ^45^, ^46^ and the higher total laser energy during the FS‐LASIK procedure could contribute both to the flare‐up of inflammatory response and the related surge of oxidative stress compared to LALEX procedure.^17^, ^42^ Although different wound sites, wound forms and stromal manipulations exist between LASIK and LALEX which may influence the reactive oxygen species level in the intrastromal region, both the corneal epithelium and aqueous humour deliver antioxidants into the corneal stroma which can regulate the oxidative stress and reactive oxygen species in corneal stroma.^47^ Consequently, the antioxidant on the ocular surface could be altered by the reactive oxygen species within the cornea.

Regarding the refractive and visual outcomes, 87% and 84% of individuals reached 20/20 UCVA of 1 month post‐operatively in the LALEX and FS‐LASIK groups, respectively. In a previous study, 71% of individuals who received LALEX surgery achieved post‐operative UCVA of 20/20, and a UCVA better or equal to 20/20 after the surgery was observed in 62% of patients received FS‐LASIK,^6^ and the visual outcome of the present study may be acceptable compared to the previous research.^6^ On the other hand, the mean sphere power plus cylinder power 1 month post‐operatively were −0.19 plus −0.60 D in the LALEX group and −0.07 plus −0.45 D in the FS‐LASIK group. As the previous studies usually regard post‐operative refractive error within 1.00 D as fair refractive outcome,^6^, ^48^ the precision of post‐operative refractive status can be considered fair. The post‐operative sphere powers between the LALEX and FS‐LASIK groups were similar, while the FS‐LASIK group illustrated a significantly lower post‐operative cylinder power than the LALEX group. This may be because the excimer laser we applied for the FS‐LASIK procedure has an iris registration function, making the centration slightly better than in the LALEX procedure. However, the difference in −0.15 D astigmatism between the two groups is lower than the basic cylinder power unit on the autorefractor, so the impact of the higher astigmatism in the LALEX group may be clinically insignificant.

There remain certain limitations in the present study. Firstly, the total case number was only 108 individuals, which is not a large study population. Secondly, antioxidants were collected by putting Schirmer strip near the conjunctiva‐limbus junction which cannot reflect the change of antioxidant levels in the cornea completely, and we did not measure the intrastromal reactive oxygen species and antioxidants concentration which will reduce the credibility of our results. However, the exploration of stromal bed after refractive surgery may elevate the risk of infection and inflammation, and thus we could only take the tear film at the nearby region. Moreover, we only traced the individuals in the present study for 1 month after the refractive surgeries. However, the recovery of visual and refractive parameters after refractive surgery may take longer^48^; thus, the antioxidant agents may keep changing during the period. Besides, the baseline TAC and AA levels were numerically different between groups in which the LALEX group showed a higher TAC and AA concentrations compared to the FS‐LASIK group. We collected the samples of FS‐LASIK and LALEX together in the same time and used one device to analyse the antioxidant expression of samples from FS‐LASIK and LALEX simultaneously. Thus, the difference of baseline antioxidant concentrations between the two groups might not resulted from our manipulation error. Still, this defect will decrease the integrity of our results despite the difference of TAC/AA between groups did not reach statistical significance, and we enrolled the baseline TAC/AA concentrations in the generalized linear mixed model to adjusted its effect. Finally, the pre‐operative sphere power in the FS‐LASIK group was significantly higher than in the LALEX group, and we did not standardize this covariate, which may contribute to some bias. Nevertheless, since the FS‐LASIK can handle the refractive error higher than −10.00 D, while LALEX cannot in clinical practice,^6^ we assume this heterogeneity as clinical reality and did not exclude those with extreme higher myopia to maintain case numbers as high as possible. Moreover, we adjusted the effect of baseline refractive statuses in the generalized linear mixed model, and thus the impact of different baseline refractive statuses between the two groups might not be prominent.

In conclusion, AA decreases significantly after the refractive surgery, while the FS‐LASIK showed a significantly higher amount of decrement compared to LALEX after the adjustment of several covariates. Furthermore, DED and high myopia patients who received FS‐LASIK demonstrated a prominent reduction in AA than the same populations received LALEX. Consequently, LALEX may be recommended for individuals with DED and high myopia considering the aspect oxidative stress. Patients who scheduled for the FS‐LASIK surgery and under higher risk of post‐operative antioxidant decrement could use the artificial tear more frequently to reduce the oxidative stress‐related damage. Besides, oral or topical antioxidants supplement could be considered for the same population. Further large‐scale prospective study to measure the change of intrastromal reactive oxygen species in different refractive surgeries and to survey the correlation between major antioxidant levels in tear film including TAC, AA and superoxide dismutase to long‐term outcomes after refractive surgeries should be undertaken.

## AUTHOR CONTRIBUTIONS

**Hung‐Chi Chen:** Methodology (equal); writing – review and editing (equal). **Shun‐Fa Yang:** Formal analysis (equal); writing – review and editing (equal). **Chia‐Yi Lee:** Conceptualization (equal); writing – original draft (lead). **Yi‐Jen Hsueh:** Data curation (equal); software (lead). **Jing‐Yang Huang:** Formal analysis (equal). **Chao‐Kai Chang:** Conceptualization (equal); methodology (equal); formal analysis (equal); data curation (equal); supervision (lead); writing – review and editing (equal); validation (lead).

## FUNDING INFORMATION

This study was supported by grants from the Chang Gung Memorial Hospital [CMRPG3M0741], [XMRPG3M0181] and the Ministry of Science and Technology, Taiwan [MOST 111‐2314‐B‐182A‐067‐MY3].

## CONFLICT OF INTEREST STATEMENT

The authors have no proprietary or commercial interest in any materials mentioned in this article.

## PATIENT CONSENT STATEMENT

The written informed consents were obtained from all participants.

## CLINICAL TRIAL REGISTRATION

This clinical trial was registered in the ClinicalTrial.gov (registration number: NCT05905237).

## Supporting information


Tables S1–S2.

## Data Availability

The data used in the present study is available from the corresponding author upon reasonable request.
