# Correction to ‘Differences in change of post‐operative antioxidant levels between laser‐assisted lenticule extraction and femtosecond laser in situ keratomileusis’

**DOI:** 10.1111/jcmm.70038

**Published:** 2024-08-27

Chen HC, Yang SF, Lee CY, Hsueh YJ, Huang JY, Chang CK. Differences in change of post‐operative antioxidant levels between laser‐assisted lenticule extraction and femtosecond laser in situ keratomileusis. J Cell Mol Med. 2024 Jan;28(2):e18069.

In AUTHOR CONTRIBUTIONS section, the text was incorrect:

‘Hung‐Chi Chen: Methodology (equal); writing—review and editing (equal). Shun‐Fa Yang: Formal analysis (equal); writing—review and editing (equal). Chia‐Yi Lee: Conceptualization (equal); writing– original draft (lead). Yi‐Jen Hsueh: Data curation (equal); software (lead). Jing‐Yang Huang: Formal analysis (equal). Chao‐Kai Chang: Conceptualization (equal); methodology (equal); formal analysis (equal); data curation (equal); supervision (lead); writing—review and editing (equal); validation (lead)’.

This should have read:

‘Hung‐Chi Chen: Methodology (equal); writing—review and editing (supporting). Shun‐Fa Yang: Formal analysis (equal); writing—review and editing (supporting). Chia‐Yi Lee: Conceptualization (supporting); writing‐original draft (lead). Yi‐Jen Hsueh: Data curation (supporting); software (lead). Jing‐Yang Huang: Formal analysis (equal). Chao‐Kai Chang: Conceptualization (lead); methodology (equal); formal analysis(equal); investigation (lead); resource (lead); data curation (lead); visualization (lead); supervision (lead); writing—review and editing (lead); validation (lead); project administration (lead); funding acquisition (lead)’.

In FUNDING INFORMATION section, the text was incorrect.

‘This study was supported by grants from the Chang Gung Memorial Hospital [CMRPG3M0741], [XMRPG3M0181] and the Ministry of Science and Technology, Taiwan [MOST 111‐2314‐B‐182A‐067‐MY3]’.

This should have read:

‘This study was supported by grants from the Chang Gung Memorial Hospital [CMRPG3M0741], [XPRPG3M0181] and the Ministry of Science and Technology, Taiwan [MOST 111‐2314‐B‐182A‐067‐MY3]’.

We apologize for this error.

