# Supplementary material for: Differences in change of post‐operative antioxidant levels between laser‐assisted lenticule extraction and femtosecond laser in situ keratomileusis
Source: J Cell Mol Med. 2023 Dec 5;28(2):e18069. doi: 10.1111/jcmm.18069 (PMC10826428; doi:10.1111/jcmm.18069)
Supplement: Supplementary file 1 — Tables S1–S2. [file JCMM-28-e18069-s001.docx]

Supplementary Table 1. The level of antioxidant before and after surgery in sex-wise subgroups

| Antioxidant | LALEX population | FS-LASIK population | P value |
| --- | --- | --- | --- |
| TAC |  |  |  |
| Male |  |  |  |
| Pre-OP | 634.35 ± 292.47 | 564.29 ± 389.32 | 0.0516 |
| Post-OP 1 week | 621.88 ± 229.61 | 562.64 ± 221.68 | 0.1034 |
| Post-OP 1 month | 659.12 ± 155.51 | 565.17 ± 150.83 | 0.0300* |
| Female |  |  |  |
| Pre-OP | 687.21 ± 232.56 | 587.54 ± 401.27 | 0.0088* |
| Post-OP 1 week | 667.30 ± 228.47 | 575.33 ± 200.68 | 0.0367* |
| Post-OP 1 month | 685.74 ± 130.02 | 592.65 ± 151.97 | 0.0114* |
| AA |  |  |  |
| Male |  |  |  |
| Pre-OP | 409.95 ± 203.68 | 355.31 ± 116.05 | 0.6884 |
| Post-OP 1 week | 353.90 ± 117.46 | 251.97 ±156.32 | 0.0025* |
| Post-OP 1 month | 362.61 ± 119.74 | 269.06 ± 172.38 | 0.0032* |
| Female |  |  |  |
| Pre-OP | 437.58 ± 175.29 | 370.12 ± 115.67 | 0.4610 |
| Post-OP 1 week | 377.12 ± 112.85 | 264.04 ± 142.93 | 0.0001* |
| Post-OP 1 month | 382.06 ± 115.63 | 288.86 ± 162.43 | 0.0007* |

LALEX: laser assisted lenticule extraction, FS-LASIK: femtosecond laser assisted-laser in situ keratomileusis, N: number, OP: operation, TAC: total antioxidant capacity, AA: ascorbic acid

* denotes significant difference between the two subgroups

Supplementary Table 2. The level of antioxidant before and after surgery in age distribution-wise subgroups

| Antioxidant | LALEX population | FS-LASIK population | P value |
| --- | --- | --- | --- |
| TAC |  |  |  |
| <35 years |  |  |  |
| Pre-OP | 673.57 ± 258.07 | 579.66 ± 386.25 | 0.0115* |
| Post-OP 1 week | 657.39 ± 217.48 | 573.17 ± 224.92 | 0.0487* |
| Post-OP 1 month | 682.01 ± 144.20 | 581.74 ± 140.99 | 0.0210* |
| ≧35 years |  |  |  |
| Pre-OP | 663.28 ± 280.36 | 571.64 ± 379.91 | 0.0127* |
| Post-OP 1 week | 648.27 ± 230.15 | 567.42 ± 228.83 | 0.0468* |
| Post-OP 1 month | 670.54 ± 149.39 | 575.45 ± 148.29 | 0.0283* |
| AA |  |  |  |
| <35 years |  |  |  |
| Pre-OP | 436.25 ± 186.92 | 369.74 ± 112.94 | 0.5007 |
| Post-OP 1 week | 376.34 ± 117.44 | 265.38 ±154.26 | 0.0007* |
| Post-OP 1 month | 379.36 ± 122.71 | 284.39 ± 170.67 | 0.0024* |
| ≧35 years |  |  |  |
| Pre-OP | 421.65 ± 208.63 | 358.17 ± 112.01 | 0.4867 |
| Post-OP 1 week | 361.23 ± 128.59 | 252.38 ± 155.97 | 0.0002* |
| Post-OP 1 month | 370.81 ± 119.35 | 271.25 ± 185.40 | 0.0008* |

LALEX: laser assisted lenticule extraction, FS-LASIK: femtosecond laser assisted-laser in situ keratomileusis, N: number, OP: operation, TAC: total antioxidant capacity, AA: ascorbic acid

* denotes significant difference between the two subgroups
